# Effect of Gold Nanoparticles in Microbial Fuel Cells Based on Polypyrrole-Modified *Saccharomyces cerevisiae*

**DOI:** 10.3390/bios14120572

**Published:** 2024-11-26

**Authors:** Kasparas Kižys, Domas Pirštelis, Inga Morkvėnaitė-Vilkončienė

**Affiliations:** Department of Nanotechnology, State Research Institute Center for Physical Sciences and Technology, 02300 Vilnius, Lithuania; kasparas.kizys@ftmc.lt (K.K.); domas.pirstelis@ftmc.lt (D.P.)

**Keywords:** *Saccharomyces cerevisiae*, yeast, wastewater, polypyrrole, 9,10-phenanthrene quinone, nanomaterials

## Abstract

Microbial fuel cells (MFCs) are a candidate for green energy sources due to microbes’ ability to generate charge in their metabolic processes. The main problem in MFCs is slow charge transfer between microorganisms and electrodes. Several methods to improve charge transfer have been used until now: modification of microorganisms by conductive polymers, use of lipophilic mediators, and conductive nanomaterials. We created an MFC with a graphite anode, covering it with 9,10-phenatrenequinone and polypyrrole-modified *Saccharomyces cerevisiae* with and without 10 nm sphere gold nanoparticles. The MFC was evaluated using cyclic voltammetry and power density measurements. The peak current from cyclic voltammetry measurements increased from 3.76 mA/cm^2^ to 5.01 mA/cm^2^ with bare and polypyrrole-modified yeast, respectively. The MFC with polypyrrole- and nanoparticle-modified yeast reached a maximum power density of 150 mW/m^2^ in PBS with 20 mM Fe(III) and 20 mM glucose, using a load of 10 kΩ. The same MFC with the same load in wastewater reached 179.2 mW/m^2^. These results suggest that this MFC configuration can be used to improve charge transfer.

## 1. Introduction

The scientific community has lately shown much interest in the development of green energy from natural sources, such as biofuel, biomass, or wastewater [1]. One of the bioelectrochemical devices is a biofuel cell (BFC), which can be used to clean wastewater by simultaneously creating power and reducing organic waste. Global wastewater production exceeds 350 billion cubic meters annually [2], which has become an enormous problem in the world. Biofuel cells are quite flexible in treating different types of wastewater [3] and they can be incorporated into current wastewater treatment plants [4]. Using biofuel cells, the efficiency of wastewater treatment can be increased, and the environmental impact can be diminished. BFC uses the catalytic or metabolic activities of enzymes, nano enzymes, or even whole cells to produce electricity [5,6]. Glucose, fructose, and sucrose, as well as complex chemical compounds, are used as biofuel.

However, microbial fuel cells (MFCs) are not widely used to generate electricity or treat wastewater because of their low performance, expensive parts and materials, and scalability issues [7]. MFCs have low efficiency due to limited charge transfer through the cell wall and membrane [8,9,10,11]. To solve this problem, conducting polymers that connect the cell cytoplasm to the extracellular matrix are used. Conducting polymers are suitable for this application because of their properties: electrical conductivity [12,13], biocompatibility [14,15], elasticity [16], and environmental stability [17]. They can modify the yeast cell wall and/or cytoplasmic membrane to increase electrical conductivity. Conducting polymers can be produced by chemical oxidative polymerization [18,19,20], electrochemical polymerization [21], enzymatic polymerization [22,23,24], and UV-induced polymerization [25]. Basic semi-conducting polymer films and polymeric nanoparticles can be synthesized using several methods [26,27]. Polypyrrole (pPy) has the highest biocompatibility with living cells and microorganisms among other conducting polymers [15,28]. Studies have shown that yeast cells can be immobilized on electrochemically generated pPy layers to create sensors that can detect chemicals that affect yeast cell metabolism [29].

*S. cerevisiae* is a biochemical reaction catalyst and possesses the capacity to exploit a diverse array of carbon sources in microbial fuel cells, such as monosaccharides, disaccharides, cellulosic biomass, glycerol, and refractory organic wastes [30]. Since *S. cerevisiae* is used in the food industry, it is also suitable for industrial waste treatment MFCs [31]. *S. cerevisiae* has high metabolic efficiency and a fast reproduction rate [32]. It can also be cultivated under simple growth conditions [33]. The yeast cell structure is complex and consists of two membranes: the plasma membrane and transmembrane electron transfer protein and the mitochondrial membrane [34]. Therefore, two redox mediators have to be used, one of which is lipophilic and can cross all cell membranes, while the second is the hydrophilic redox mediator, which transfers electric charge from the extracellular environment toward the electrode [29]. In our prior research, we demonstrated the potential use of PQ as a redox mediator to assess yeast viability [8,29,35]. It exhibited a significantly higher electrochemical signal than p-benzoquinone, 2,6-dichlorophenolindophenol sodium salt hydrate, and 10-phenanthroline-5,6-dione. According to the findings, it was anticipated that PQ could be a good choice for designing a yeast-based MFC to enhance the transfer of electrical charge across the yeast membrane and cell wall toward the electrode. 

There is a strong tendency for nanoparticle applications in biology [36], industrial chemistry [37], electrochemical sensing devices [38], and bioelectrochemistry [39]. Gold nanoparticles are known as good candidates for achieving higher charge transfer [40,41]. Among all possible applicable nanoparticles, 10 nm gold sphere nanoparticles were chosen for our research due to their low cytotoxicity when bigger size nanoparticles are used [42] at lower concentrations [43]. 

Our research aimed to develop an MFC consisting of yeast modified by pPy and gold nanoparticles to increase charge transfer and power density.

## 2. Materials and Methods

### 2.1. Materials

Potassium ferricyanide and potassium ferrocyanide (>99%) were purchased from Carl Roth, Karlsruhe, Germany. Whatman^®^ Cyclopore Track Etched Membrane, 9,10-Phenantrenequinone, YPD broth, pyrrole (98%), D-(+)-glucose (99%), citrate-buffered 10 nm gold sphere nanoparticles (OD = 1), and phosphate-buffered saline (PBS) tablets were purchased from Merck, Darmstadt, Germany. Yeast cell strain *S. cerevisiae* Y00000 (BY4741 Matαhis 3Δ1 leu 2Δ0 met 15Δ0 ura 3Δ0) was obtained from EUROSCARF (Frankfurt, Germany). Graphite rod electrodes (99.9995%) were purchased from Thermo Fisher, Waltham, MA, USA. Ethanol (98%) was purchased from Spiritus Vilnensis, Vilnius, Lithuania.

**Figure 1:**
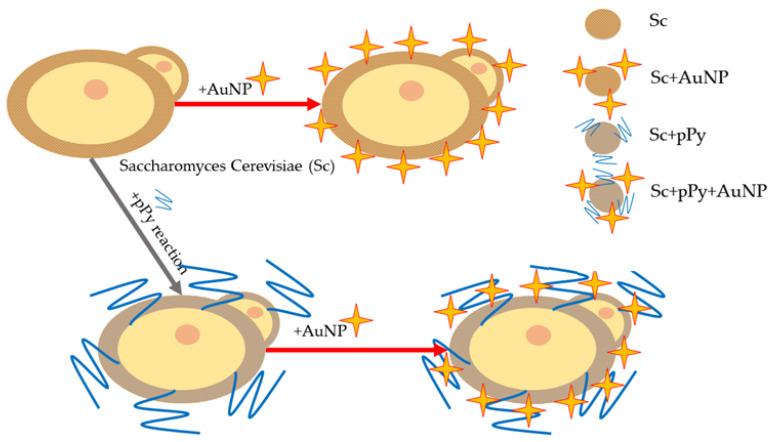
*S. cerevisiae* (Sc) modification scheme. pPy—polypyrrole within yeast cell wall. AuNP—gold nanoparticles, 10 nm spheres.

*S. cerevisiae*, batch Y00001 cultures were prepared by using a laminar cabinet and thermo-regulated shaker. Yeast growth was induced using a YPD broth (yeast extract peptone dextrose solution) consisting of a glucose, yeast extract, and peptone solution in deionized water in a +30 °C incubator, shaking at 200 rotations per minute. The YPD broth was inoculated with cultured an *S. cerevisiae* solution that was prepared earlier from a deep-frozen library sample; 100 µL of solution was used each time. Growth continued for 24 h while preparing each batch. No batch was used longer than 48 h after stopping the growth process. Each time, after stopping the growth process, the yeast solution washing process with PBS took place. The procedure consisted of delivering the yeast solution to Eppendorf tubes, and centrifugation was carried out for 2 min at 3000 rpm at room temperature. Then, the supernatant was discarded from each tube, PBS was reintroduced to the precipitate, the solution was homogenized with an automatic micropipette, and the centrifugation process was repeated. The same process was repeated once more and, after the centrifugation and discarding of the supernatant for the last time, all the tube contents were diluted to a 1:1 mass ratio with PBS,. We considered the PBS density to be 1 g/L, securing the standard yeast solution of 1 g/mL. This solution was used for the experiment itself and the modification. The polypyrrole modification was performed according to a [44] patent recipe with some changes. The main ingredient, pyrrole, was introduced into the solution, which consisted of potassium ferrocyanide (II), glucose, and yeast solution, all in PBS at the volumetric ratio represented in Table 1. Gold nanoparticles were added in Ppy-modified and non-modified yeast (Figure 1).

Graphite rod electrodes, 3.05 mm diameter × 30 mm long, were polished using various sanding papers with grit sizes ranging from 300 to 2000, cleaned with 96% methanol and deionized water, and dried. The working surface area was 7.302 mm^2^. The graphite electrode was placed in a silicone tube.

We used 9,10-phenanthrenequinone (PQ) as the lipophilic mediator; it was dissolved in 98% ethanol, and a 2 µL 3 mM drop was deposited onto the graphite electrodes. Furthermore, every electrode was modified by drop-casting 2 µL of 1 g/L of yeast solutions, which were either non-modified or modified by gold nanoparticles and polypyrrole. 

Gold sphere nanoparticles measuring 10 nm were used for additional modifications. The ratio for modification was two parts yeast solution per one part of nanoparticle solution.

### 2.2. Methods

#### 2.2.1. Cyclic Voltammetry

A three-electrode setup consisted of a PQ and yeast-modified graphite working electrode, an Ag/AgCl reference electrode, and a platinum counter electrode (Metrohm AG, Herisau, Switzerland). The electrochemical cell was filled with PBS containing 20 mM of potassium ferricyanide (K_3_[Fe(CN)_6_]) and 20 mM of glucose. Experiments started after 20 min.

The peaks of the CV were evaluated using a modified Hill function with an offset:

(1) $$j=\frac{C^{n}}{k^{n}+C^{n}}$$

where *j* is the current density, *C* is the glucose concentration, *k* is a constant that determines glucose concentration at half of the maximum current density registered, and *n* is the Hill coefficient.

#### 2.2.2. MFC Measurements

The experiments were carried out with graphite rod electrodes: one was modified with the yeast and/or corresponding chemicals under the membrane as an anode; the other was an unmodified graphite rod, cleaned with 96% methanol and dried, used as a cathode. The media were prepared in a phosphate buffer saline (PBS) solution with 20 mM K_3_[Fe(CN)_6_] and 20 mM glucose. Before every experiment, the working electrode was incubated in the solution for 20 min. The environment for the experiment was aerobic. All experiments were conducted at least four times, with each trial using a newly made electrode. After an incubation period of 20 min, potential measurements were done at least four times for each load. Resistance was gradually lowered in a logarithmic scale, from 1000 kΩ to 1 Ω.

## 3. Results

**Figure 2:**
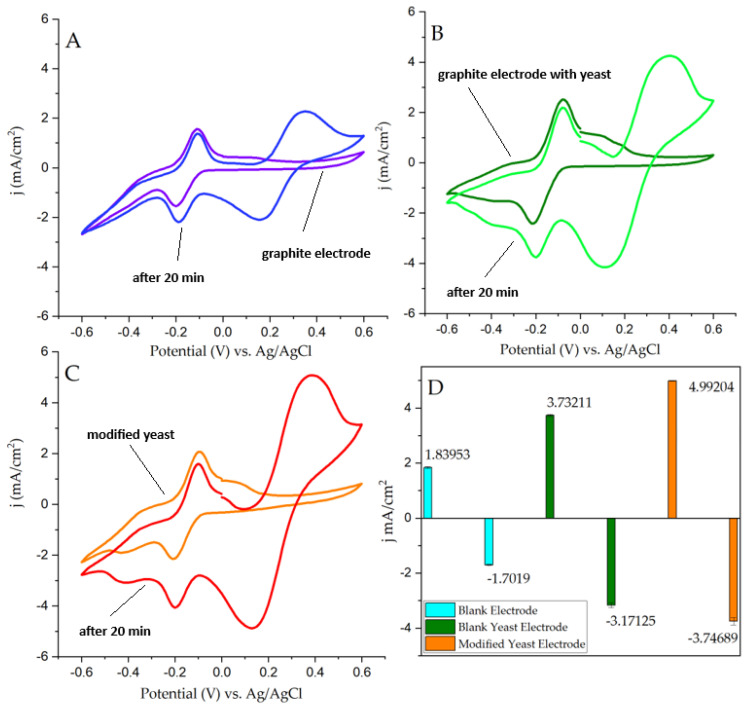
Cyclic voltammetry of three types of PQ−covered graphite electrodes: (**A**) Blank, without other additives; (**B**) BlankYeast, only with bare *S. cerevisiae* and PCTE membrane with 3 µm pores; and (**C**) ModYeast, 200 mM polypyrrole-modified *S. cerevisiae* with the same PCTE membrane. (**D**) CV peaks.

To determine the effect of PpY on yeast, cyclic voltammetry experiments were performed without yeast (A), with unmodified yeast (B), and with yeast in which the cell wall was modified (C) with 200 mM pyrrole (Figure 2). Measurements were performed before and after yeast incubation for 20 min in the working solution. PQ peaks stayed almost the same, while potassium ferrocyanide peaks increased with time. From the registered CVs (Figure 2), the current density increased using PpY-modified yeast compared to non-modified or control (without yeast). Comparing redox peaks (Figure 2D) between the empty electrode and regular yeast MFC, the acquired current density on the oxidative peak was increased twice with yeast, from 1.806 mA/cm^2^ to 3.759 mA/cm^2^, and on the reductive peak, from 1.680 mA/cm^2^ to 3.258 mA/cm^2^. After comparing bare yeast and polypyrrole-modified MFCs, modified electrode oxidative peaks showed a 33% increase in current density, reaching 5.008 mA/cm^2^ in total (Figure 2C). Reductive peaks reached 3.606 mA/cm^2^—an increase—all in comparison with the blank electrode (Figure 2A) and non-modified yeast electrode (Figure 2B) CV peaks.

**Figure 3:**
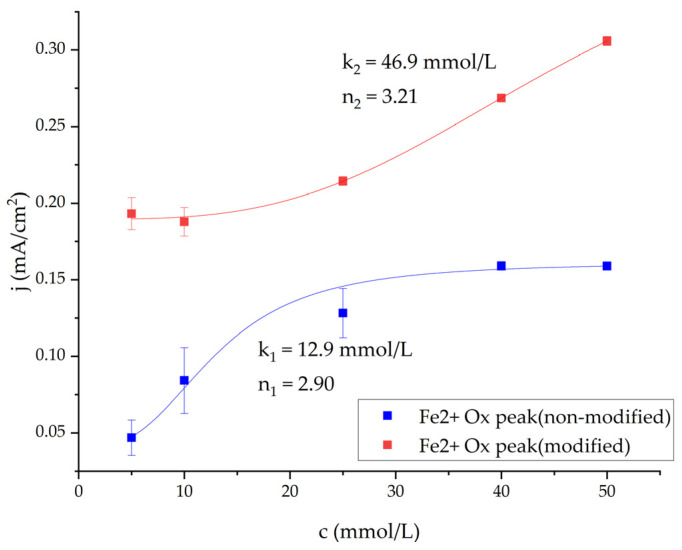
Current density dependencies on glucose concentration extracted from [Fe(CN)_6_]^4^—peaks in CV. We used 200 mM pyrrole-modified and non-modified yeast in the experiments and fitted them using Hill’s equation (Equation (1)).

The obtained CV data were fitted using the modified Hill function (Equation (1)) to plot and predict the current density response of MFCs to the changes in glucose concentration (Figure 3). The resulting Hill coefficient n value for both systems (utilizing either modified or non-modified yeast cells) was greater than 1 (Figure 3); this indicates positive cooperativity between substrate and enzyme molecules, meaning the addition of glucose enhances the reaction of more substrate molecules. Additionally, the k value, which represents the concentration of glucose in the system where half of the current is produced, is lower in the non-modified yeast system (k = 12.9 mmol/L); this indicates that unmodified yeast possesses a greater affinity for the substrate, unlike the modified yeast (k = 46.9 mmol/L). Even though PpY-modified yeast may possess inferior substrate affinity, the current density curve of the system demonstrates better performance, indicating that the system has a greater capacity for power generation. Therefore, more electrical energy can be generated using modified yeast.

**Figure 4:**
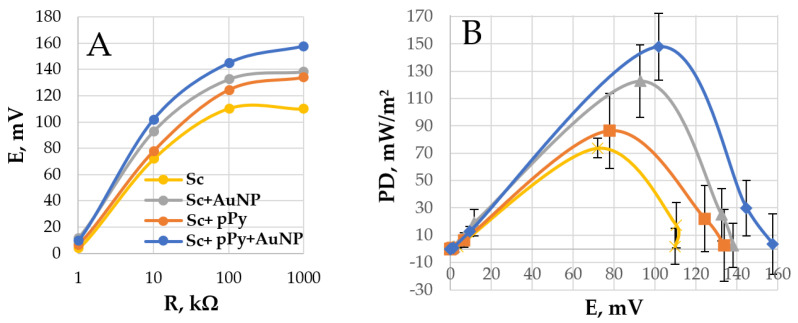
(**A**) Potential dependencies on load. (**B**) Power density (PD) dependencies on potential. Measurements were performed in the PBS solution with glucose (20 mM) and K_3_[Fe(CN)_6_] (20 mM). Electrodes were covered with PQ and *S. cerevisiae* with various modifications shown in Figure 1.

Furthermore, yeast was modified with 50 mM pyrrole and 10 nm gold nanoparticles added in a two-part yeast to one-part nanoparticle solution ratio. Maximum power densities were obtained using a 10 kΩ load at 0 min (Figure 4). Bare yeast showed the lowest power density in this experiment—on the load of 10 kΩ, the MFC reached 73.7 mW/m^2^ (Figure 4A). In comparison, a power density of 86.5 mW/m^2^ peaked at the same load for 50 mM pyrrole-polymerized *S. cerevisiae* cell wall MFC. The addition of 10 nm gold nanoparticles into the composition of MFCs showed a rise in potential, and the calculations determined that the power density of MFCs of bare yeast with such an add-on reached 122.8 mW/m^2^, which is 66.6% higher than without the addition of nanoparticles. Then, the MFCs of a modified yeast cell wall with the same nanoparticles were studied in the same manner; they reached a power density of 147.8 mW/m^2^, twice the peak power density of the bare yeast anode (Figure 4B).

**Figure 5:**
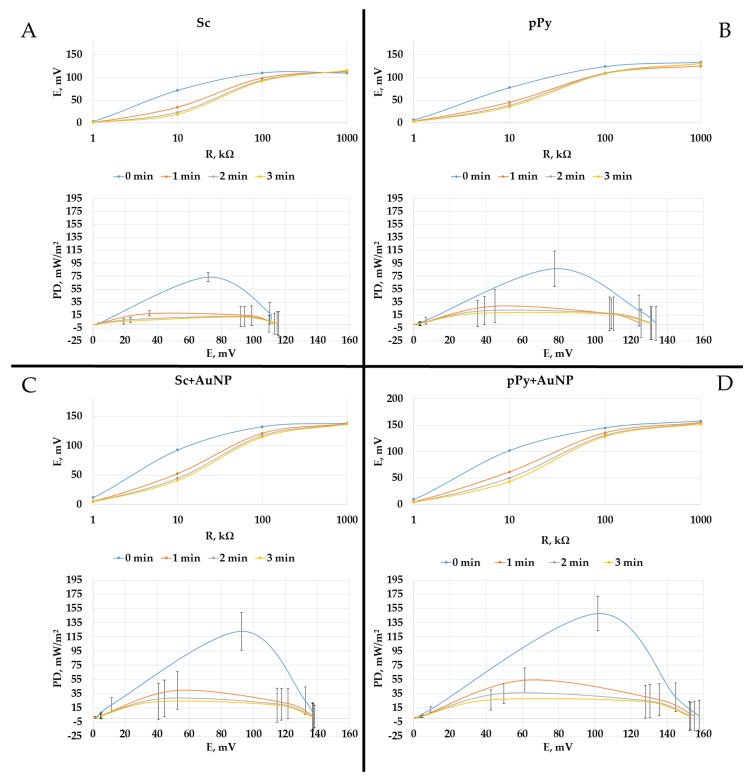
Potential vs. load and power density (PD) vs. potential dependencies, measured in time with glucose (20 mM) and potassium ferricyanide (20 mM) in PBS. (**A**) Bare yeast MFC. (**B**) Polymerized yeast cell wall MFC. (**C**) Yeast modified with nanoparticles. (**D**) Yeast modified with pPy and nanoparticles.

MFC power density dependencies on different loads were measured every minute for 3 min (Figure 5). Power density dropped with every minute. However, the highest power density was observed when yeast was modified with pPy and gold nanoparticles.

**Figure 6:**
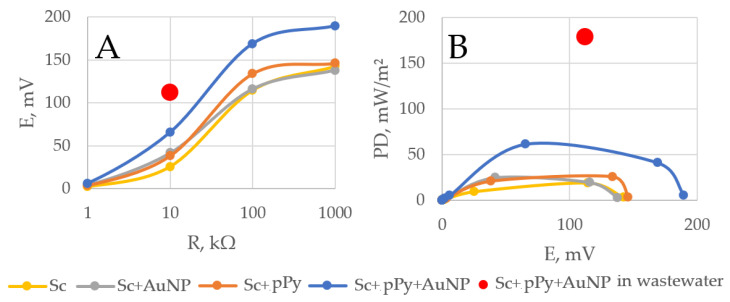
(**A**) Potential dependencies on load. (**B**) Power density (PD) dependencies on potential. Measurements were performed in the glucose (20 mM) and K_3_[Fe(CN)_6_] (20 mM) solution. The red dot represents a sample of UAB’s “Vilniaus Vandenys” wastewater facility.

The best-performing MFC was determined to be the one with a 50 mM pyrrole-polymerized yeast cell wall with an add-on of 10 nm Au nanosphere particles on a polished graphite rod covered with PQ and an all-topping 3 µm pore PCTE membrane. With this configuration, we measured actual wastewater under a load of 10 kΩ. After a 3 min evaluation, the power density reached 179.2 mW/m^2^ (Figure 6, the red dot). In comparison, the power density outcomes of the previous experiments after 3 min and with 10 kΩ load were 9.2 mW/m^2^ for bare yeast MFC, 20.6 mW/m^2^ for polypyrrole yeast MFC, 24.6 mW/m2 for bare yeast with nanoparticles, and 61.1 mW/m^2^ with MFC in PBS with glucose and potassium ferricyanide solution.

## 4. Discussion

In this study, we investigated the application of a conductive polymer, polypyrrole, in conjunction with 10 nm gold nanoparticles for modifying *S. cerevisiae* (Sc) cells for application in a microbial fuel cell. We demonstrated an increase in current density when utilizing yeast cells that were modified with 50 mM of pyrrole from 3.76 mA/cm^2^ for unmodified Sc cells to 5.01 mA/cm^2^ for pPy modified Sc cells (Figure 2). Additionally, the performed power density measurements have shown that AuNP- and pyrrole-modified yeast cells significantly improve the power density (using a 10 kΩ resistor) of the MFC—9.2 mW/m^2^ for bare yeast MFC, 20.6 mW/m^2^ for pPy modified yeast MFC, 24.6 mW/m^2^ for bare yeast with nanoparticles, and maximum of 61.1 mW/m^2^ for the AuNP-pPy-modified yeast cells in 20 mM glucose and K_3_[Fe(CN)_6_] solution. Finally, power measurements with the AuNP-pPy-modified yeast MFC were performed in a wastewater sample, and the registered power density at 10 kΩ was 179.2 mW/m^2^ (Figure 6). The power density increased because of organic compounds in wastewater, such as carbohydrates, proteins, and fats, which serve as nutrients for yeast.

The performance of pyrrole-modified *S. cerevisiae* in MFCs, as demonstrated in this research, is consistent with previous studies regarding the power outputs. A microbial fuel cell utilizing the 9,10-phenanthrenequinone and potassium ferricyanide redox mediator system deposited on a polished carbon electrode and consumer-grade yeast cells demonstrated higher, but similar, power density results of 22.2 mW/m^2^ [8]; an electrode constructed using the same materials but incorporating pyrrole-modified Sc cells was shown to generate a power density of 47.12 mW/m^2^ at 129 mV [29]. Compared to these results, our constructed MFC consisting of laboratory-grade yeast cultures has shown inferior power generation; nevertheless, using gold nanoparticles in conjunction with pyrrole modification of the yeast cells resulted in a greater increase in the power density of the system—61.1 mW/m^2^. This coincides with the fact that the usage of gold nanoparticles has previously been shown to improve the charge transfer between yeast cells and the electrode by bridging the gaps between conductive materials in the system and the microorganisms—a yeast MFC system composed of polyethyleneimine-modified carbon felt with gold nanoparticles prepared with 715 μM mercaptobenzoic acid achieved a power density of 2771 ± 569 mW/m^2^ [45]. In general, the usage of nanomaterials (carbon nanotubes, graphene, and metal nanostructures) has been shown to significantly increase the power density of the MFCs [45,46,47,48,49,50,51] either by expanding the electrochemically active surface of the electrode, providing additional attachment sites for the microbes, or improving the extracellular charge flow. Furthermore, the application of polypyrrole demonstrated a substantial increase in power density, indicating improved extracellular charge transfer. This finding is consistent with numerous studies involving the application of conducting polymers in MFCs [29,45,47,49,51,52,53,54,55,56,57,58] (Table 2). 

Of particular interest was the synergistic effect observed when combining two different conductive materials—PPy and AuNPs—which produced the highest power density output. Comparable results have also been documented, wherein a composite of biogenic gold nanoparticles and multiwalled carbon nanotubes used in conjunction with a mixed culture of exoelectrogens provided the highest power outputs and highest microbe affinity to the surface compared to unmodified anode systems [51].

When considering the performance of our constructed MFC for use in wastewater treatment, the generated power density (179.2 mW/m^2^) is sufficient compared to other similar designs, such as a single-chamber MFC with a stainless steel anode modified with PPy, which in conjunction with a mixed culture of electrogenic bacteria achieved a maximum power density of 1190.94 mW/cm^2^ in anaerobic granular sludge from fruit wastewater plant [52] or a dual-chambered MFC based on PEDOT:PSS and a graphene-modified graphite anode which incorporated *G. Oxydans* to produce a power density in municipal wastewater of 82 mW/cm^2^ [55]. A significant increase in open-circuit potentials and elevated measured power densities when testing our designed MFC system in the wastewater sample could be attributed to the presence of vast amounts of multiple types of saccharides in the said sample, which could facilitate faster metabolic activity. Moreover, the wastewater sample may have an optimal concentration of naturally occurring redox mediators, which could improve the charge transfer from the microorganisms to the electrode surface. 

There are two key limitations to the research we conducted. The first one is using *S. cerevisiae* as the main carbohydrate catalyst for energy generation in our system; since the catalysis of glucose happens deep inside the cell and the mechanism of charge transfer is not direct, the electricity generation process requires a system of mediating agents. By using prokaryotic microorganisms for MFCs, such as bacteria, which lack basic eukaryotic organelles and therefore conduct metabolism on their cell wall, the charge transfer limitations are lowered. Furthermore, most bacteria can transport electricity to the electrode by utilizing their long structural appendages, which are called nanowires [59]. 

In addition to the choice of the microorganism for the fuel cell system, several structural design solutions could be implemented to improve the power output of the MFC. One of the main structural elements in the system that could be improved is the geometry and material of the anode [60,61]; a more porous material, such as carbon felt, could be used for a larger electroactive surface area. Meanwhile, graphene, reduced graphene oxide, or carbon nanotubes could be used to enhance the conductivity of the electrode. Furthermore, the cathode material could be modified to optimize the reduction reaction, thus removing any limitations arising from the slow proton reduction reaction. A proton exchange membrane and a different geometry of the cell, e.g., double chamber vs. single chamber, could also be considered when designing an improved microbial fuel cell.

## Figures and Tables

**Table 1:** Preparation of modified yeast recipe.

Component	Initial Concentration	Mixture Concentration
Yeast	1 g/mL	0.2 g/mL (20%)
Glucose	1 M	400 mM (40%)
Potassium ferrocyanide (II)	0.4 M	80 mM (20%)
PBS ^1^	0.1 M	0.1 M
Pyrrole	98%	50 mM/0.7% or 200 mM/2.8%

^1^ Main solvent, consisting of pH 7.4 standard PBS solution in water.

**Table 2:** MFC power density.

Microbe/Anode Material	Modification	Substrate	Control PD (mW/m^2^)	Max. PD (mW/m^2^)	Ref.
Mixed culture/graphite	FeNP’s	Acetate	997	1856	[46]
Mixed culture/carbon cloth	PANI-graphene	Acetate	454	884	[47]
*S. Cerevisiae* C/glassy carbon	CNT’s	Glucose	138	344	[48]
*Shewanella xiamenensis*/BC-PANI	TiO_2_	Glucose	137.4	179.4	[49]
*S. Cerevisiae*/carbon felt	AuNP-PEI	Glucose	381	2771	[45]
Mixed culture/carbon cloth	BioAu-MWCNT	Glucose	114.24	178.34	[50]
*S. Cerevisiae*/carbon felt	PEI-FeMnNP	Glucose	380	5800	[51]
Mixed culture/stainless steel	PPy	Wastewater	40.59	1190.94	[52]
Mixed culture/carbon brush	PPy-CMC-CNT	Acetate	683	2970	[53]
*S. Cerevisiae*/graphite rod	PPy	Glucose	38.8	47.12	[29]
Mixed culture/graphite plate	PANI	Potato powder/soybean powder	91.5	256.4	[54]
*Gluconobacter Oxydans/*graphite rod	PEDOT:PSS-graphene-Nafion	Wastewater	-	82	[55]
Mixed culture/carbon felt	PEDOT:PSS-TEG	Glucose	0.8	68.7	[56]
Mixed culture/carbon veil	PEDOT:PSS	Urine	0.4305	0.5351	[57]
Mixed culture/nickel foam	MgCoO_2_- PEDOT:PSS	Wastewater	197.6	494	[58]
*S. Cerevisiae*/graphite rod	PPy-AuNP’s	Glucose Wastewater	9.2 -	61.1 179.2	This work

FeNP, iron carbide nanoparticles; PANI, polyaniline; BioAu, biogenic gold nanoparticles; CNT, carbon nanotubes; MWCNT, multiwall carbon nanotubes; BC, biocellulose; PEI, polyethiylenimine; FeMnNP, iron-manganese nanoflowers; PPy, polypyrrole; CMC, carbomethyl cellulose; PANI, polyaniline; PEDOT:PSS, poly(3,4-ethylene dioxythiophene):poly(4-styrene sulfonate); TEG, thermally expanded graphite.

## Data Availability

The original contributions presented in the study are included in the article, further inquiries can be directed to the corresponding author.
